# Curcumin relieved the rheumatoid arthritis progression via modulating the linc00052/miR-126-5p/PIAS2 axis

**DOI:** 10.1080/21655979.2022.2066760

**Published:** 2022-04-27

**Authors:** Jianwei Xiao, Xu Cai, Weijian Zhou, Rongsheng Wang, Zhizhong Ye

**Affiliations:** 1 Department of Rheumatology and Immunology, Shenzhen Futian Hospital for Rheumatic Diseases, No.22 Nonglin Road, Shenzhen 518000, China; 2 Department of Rheumatism, Yunnan Provincial Hospital of Traditional Chinese Medicine. NO.120 Guanghua Street, Wuhua District, Kunming City, Yunnan Province, 650000, China; 3 Department of Rheumatism, Shanghai Guanghua Hospital of Integrated Traditional and Western Medicine, Shanghai, 200052, China

**Keywords:** Curcumin, rheumatoid arthritis, linc00052, protein inhibitor of activated STAT 2

## Abstract

Curcumin, with its antioxidant, anti-inflammatory, and antitumor properties, is widely used in the treatment of bone disorders, including rheumatoid arthritis (RA). We investigated the effects of curcumin on fibroblast-like synoviocytes in RA and its underlying mechanism. mRNA and microRNA (miRNA) expression levels were determined using reverse transcription-quantitative polymerase chain reaction. Cellular functions were detected using cell counting kit-8, 5-ethynyl-2’-deoxyuridine, Transwell, and flow cytometric assays. Enzyme-linked immunosorbent assay was performed to measure the cytokine release. Western blotting was used to determine the protein expression levels. An *in vivo* assay was performed to verify the role of linc00052 in RA. Curcumin promoted apoptosis and inhibited the growth, migration, and invasion of RA fibroblast-like synovial (RAFLS) cells. Curcumin treatment suppressed the inflammatory response of RAFLS cells. Moreover, curcumin increased linc00052 levels, and linc00052 knockdown reversed the effects of curcumin. Additionally, linc00052 functioned as a competing endogenous RNA to upregulate the expression of the protein inhibitor of activated STAT 2 (PIAS2) by sponging miR-126-5p. Curcumin inhibited the Janus kinase 2 (JAK2)/signal transducer and activator of transcription 3 (STAT3) signaling pathway. *In vivo* assays showed that curcumin decreased the arthritis score and improved inflammatory infiltration and synovial cell proliferation. These results reveal that curcumin protects against RA by regulating the inc00052/miR-126-5p/PIAS2 axis through JAK2/STAT3 signaling pathway.

## Graphical abstract

**Figure:**
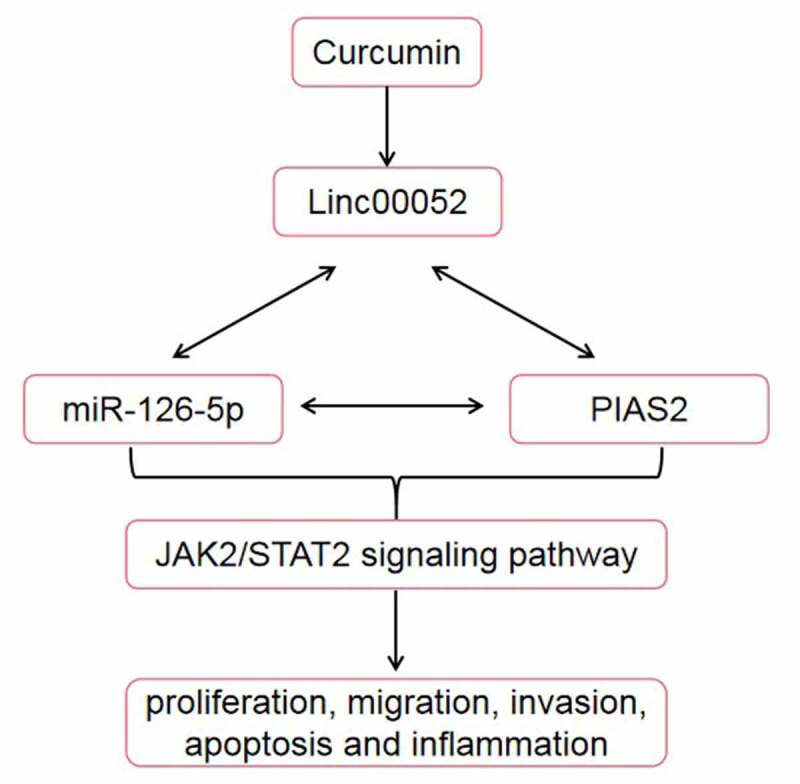

## Highlights

- Curcumin alleviates the severity of arthritis.
- Expression of linc00052 is decreased in RA.
- Knockdown of linc00052 reverses the effects of curcumin

## Introduction

Rheumatoid arthritis (RA) is a progressive chronic autoimmune disease [1,2]. RA increases the risk of disability. It constitutes a heavy social burden and significantly affects the patients’ lives [2,3]. Currently, there is no cure for RA due to the poor understanding of its pathology. RA lesions consist of atypical synovial hyperplasia. Fibroblast-like synoviocytes (FLSs) secrete proinflammatory factors, chemokines, cathepsin, and matrix metalloproteinases to break down the cartilage and extracellular matrix, which induces the infiltration of inflammatory cytokines and growth factors [4]. Therefore, RA fibroblast-like synovial (RAFLS) cells may be the Achilles heel that can suppress the cellular functions of RA.

**Figure 1.**
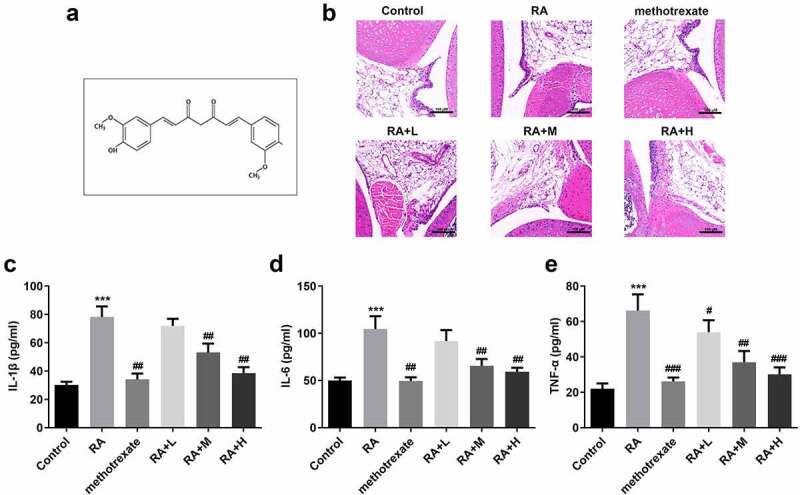
Curcumin alleviates the severity of arthritis in mice with collagen-induced arthritis. (a) Structural formula of curcumin. (b) Hematoxylin and eosin staining and Inflammation score of RA mice. (c-e) Levels of interleukin (IL)-1β, IL-6, and tumor necrosis factor (TNF)-α in collagen-induced RA mice. ***P < 0.01 vs. CON; ^##^P < 0.01, ^###^P < 0.001 vs. RA; CON, control; RA, rheumatoid arthritis; Cur, curcumin.

Curcumin (C_21_H_20_O_6_, Figure 1a) is an extract obtained from the roots of plants belonging to the *Araceae* and *Zingiberaceae* families. Curcumin possesses various pharmacological properties and shows only a few side effects [5,6]. Nurfina et al. [7] demonstrated that the 4-hydroxy group is the functional group responsible for the anti-inflammatory activity of curcumin. Previous studies have demonstrated the role of curcumin in the occurrence and development of RA [8–11]. However, the underlying mechanism remains unclear.

Long non-coding RNAs (lncRNAs), a class of endogenous non-coding RNAs, are crucial regulators of biological processes, such as proliferation, apoptosis, migration, and the inflammatory response. Several ncRNAs are considered promising biomarkers for RA diagnosis and treatment [12]. Previous studies have demonstrated the oncogenic roles of linc00052 in various cancers [13,14]; however, its role in RA remains unclear.

Therefore, in the present study, we investigated the effects of curcumin on RA both *in vivo* and *in vitro*. Furthermore, an in vivo RA model was constructed via collagen induction. In addition, we explored the underlying molecular mechanisms of RA. We hypothesized that curcumin may relieve RA by upregulating linc00052/miR-126-5p/PIAS2 axis and inhibiting the JAK2/STAT3 signaling pathway.

## Materials and methods

### Animal modeling and histopathological examination

We established an RA animal model. Briefly, 40 4–6-week-old DBA/1 J mice (half male and half female) were reared at 20–25°C and 55–65% relative humidity. During the feeding period, rats were allowed to drink and eat freely. After seven days of adaptive feeding, the mice were injected multiple times with bovine type II collagen and Freund’s adjuvant in their tails according to a previous study [15]. For boosted immunization, DBA/1 mice were re-injected after 21 d. Two days later, 20 successfully modeled mice were randomly divided into four groups: RA, RA + methotrexate positive group, RA + 25 μL curcumin, RA + 50 μL curcumin, and RA + 100 μL curcumin groups. On the 49th day, the rats were sacrificed by dislocating the cervical spine, and joint synovial tissues were collected. Tissues were immersed in paraformaldehyde (4%/48 h) and embedded in paraffin. Histopathological analysis was performed using hematoxylin and eosin staining as previously described [16]. The arthritis scores were categorized as follows: 0, typical; 1, apparent redness and swelling in the ankle/finger; 2, two joints are involved; 3, two or more joints are involved; and 4, severe arthritis of the entire paw and all fingers.

This study was approved by the Approval of Animal Care Board of Shenzhen Futian Hospital for Rheumatic Diseases, ethical code: FS202012002.

### Cell culture and treatment

RAFLS cells were provided by the Cell Bank of Chinese Academy of Sciences (Shanghai, China). The cells were cultured in the Dulbecco’s modified Eagle medium containing medium containing 10% fetal bovine serum at 37°C in a 5% CO_2_ incubator [17]. The cells were collected for further experiments.

### Enzyme-linked immunosorbent assay (ELISA)

The release of tumor necrosis factor (TNF)-α, interleukin (IL)-1β, and IL-6 was detected using an ELISA kit provided by Vazyme Biotech Co. Ltd. (Nanjing, China) [18].

### Reverse transcription-quantitative polymerase chain reaction (qPCR) and cell transfection

RAFLS cells were cultured and treated with curcumin at indicated concentrations. The cells were then collected and lysed in the Trizol reagent to extract the total RNA. RNA was reverse-transcribed into cDNA using a cDNA synthesis kit (Takara, China). The qPCR program was initiated as follows: one cycle at 95°C for 10s and 45 cycles at 95°C for 5s and 60°C for 30 . Results were calculated using the 2^−ΔΔCq^ method [19]. The sequences of the primers used in this study were as follows:

Linc00052 forward, 5**′**-CCTGAAGTTTCTCCATGAATTGTG-3**′** and reverse, 5**′**-GAGGGAGGGAGACTGAGATT-3′;

miR-126-5p forward 5′-GGTATAATCCGCCGCTTAGCTGCC-3′ and reverse 5′-GTGCAGGGTTGCAAGGT-3′;

PIAS2 forward, 5′-ATCCACGAACTCTTGAAGGACT-3′ and reverse 5′-TGTGGGCTTAGTATCTTGAAGCA-3′;

GAPDH forward, 5′-TGTGGGCATCAATGGATTTGG-3′ and reverse 5′-ACACCATGTATTCCGGGTCAAT-3′.

For cell transfection, small interfering RNA of linc00052 (si-linc00052) and si-RNA negative control were obtained from RiboBio (China) [20]. According to the Invitrogen Lipofectamine 2000 Transfection Reagent protocol, 100 nmol/L of si-linc00052 or si-RNA negative control was transfected into RAFLS cells.

### Transwell assays

Transwell assays were performed to measure the cell migration and invasion abilities [21]. The lower chamber contained 400 μL of medium containing 10% fetal calf serum, and 200 μL of cell suspension (with Matrigel for invasion and without Matrigel for migration) was added to the upper chamber. After 24 h, the cells at the bottom chamber of the Transwell membrane were fixed with 4% paraformaldehyde and stained with 0.5% crystal violet. Finally, a microscope was used to observed the migrated or invaded cells.

### Western blotting

Total protein was collected from the cells. The protein concentration was calculated. Proteins were isolated using 10% sodium dodecyl sulfate-polyacrylamide gel electrophoresis and transferred onto polyvinylidene fluoride membranes. The membranes were blocked with 10% nonfat milk. Next, the membranes were incubated with primary antibodies followed by a secondary antibody. The bands were visualized using an ECL kit and analyzed using the ImageJ software [22].

### CCK8 and EdU assays

The cell viability was assessed with CCK8 and EdU assays [23]. The cells were seeded into a 96-well plate in the presence of various curcumin concentrations for 12, 24, 48, and 72 h. Then, 10 μL/well of CCK-8 reagent was added and the cells were cultured for 2 h. Absorbance was measured at a wavelength of 450 nm. EdU assay was performed following the BeyoClick EdU-647 kit protocol (Beyotime Institute of Biotechnology).

## Flow cytometry

The cells were resuspended in pre-cooled phosphate-buffered saline and centrifuged twice. Next, 1 mL of binding buffer was used to suspend the cells, followed by the addition of 5 μl of fluorescein isothiocyanate and 5 μl of propidium iodide [24]. The cells were then incubated at 25°C for 15 min in the dark, and the apoptosis rate was detected using flow cytometry.

## Statistical analysis

Data were analyzed using SPSS 20.0 and expressed as the mean ± standard deviation. Differences were analyzed using Student’s *t*-test and analysis of variance followed by Duncan’s multiple range test. P < 0.05 was considered to be statistically significant.

## Results

This study revealed that curcumin suppressed the inflammatory response and synovial hyperplasia, while promoting the apoptosis of RAFLS cells via the linc00052/miR-126-5p/PIAS2 axis. These findings may aid in the development of a novel therapeutic strategy for the treatment of RA.

### Curcumin alleviates the severity of arthritis in mice with collagen-induced arthritis

Figure 1a shows the molecular structure of curcumin. Histological analysis revealed that the tissue structure in the CON group was relatively complete, and there was no inflammatory cell infiltration. In the RA group, the synovial tissues of mice showed epithelial stratified hyperplasia, accompanied by hemorrhage, irregular cell arrangement, and high inflammatory cell infiltration; however, the synovial tissue damage and infiltration of inflammatory cells were significantly improved in the curcumin group (Figure 1b). Additionally, curcumin treatment decreased RA scores in a dose-dependent manner (Figure 1b), and remarkably reduced the levels of proinflammatory cytokines (Figure 1c–e). Besides, the the high concentration of curcumin treatment reached the treatment level of positive control (methotrexate treatment).

### Curcumin induces the degradation of RAFLS cells

**Figure 2.**
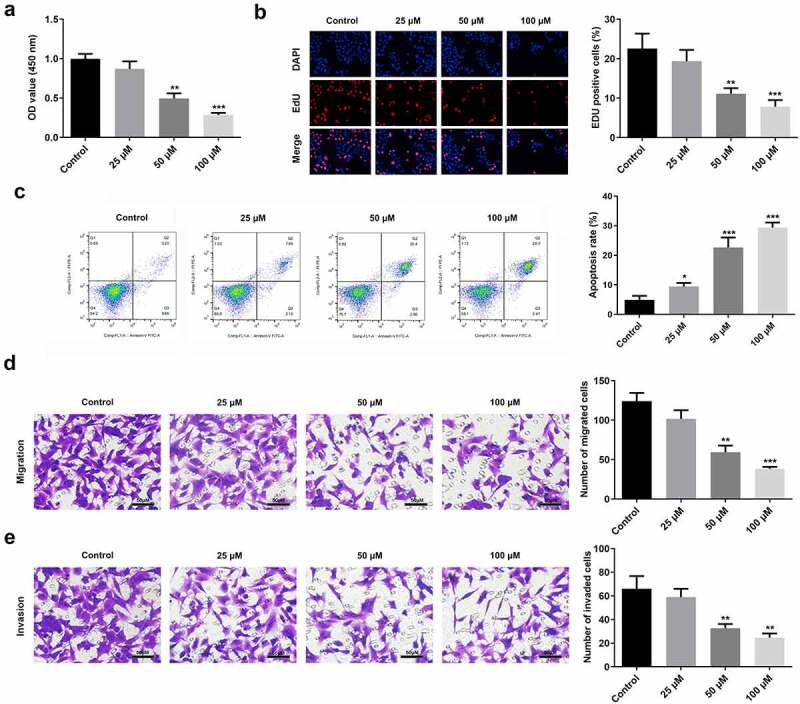
Curcumin promotes cell apoptosis and suppresses cell proliferation, invasion, and migration. (a-b) Cell counting kit (CCK)-8 and 5-ethynyl-2’-deoxyuridine (EdU) assays were conducted to determine the cell proliferation of rheumatoid arthritis fibroblast-like synovial (RAFLS) cells treated with gradient concentrations of curcumin. (c) Apoptosis rate was measured using the Flow cytometry apoptosis assay. (d-e) Migrated and invaded RAFLS cells were pictured and counted. **P < 0.01, **P < 0.01, ***P < 0.01 vs. Control. Cur, curcumin.

Curcumin inhibited the viability and proliferation of RAFLS cells in a dose-dependent manner (Figure 2(a,b)). It also increased the apoptosis rate of RAFLS cells (Figure 2c), while inhibiting their migration and invasion (Figure 2(d,e)).

### Curcumin promoted linc00052 expression

**Figure 3.**
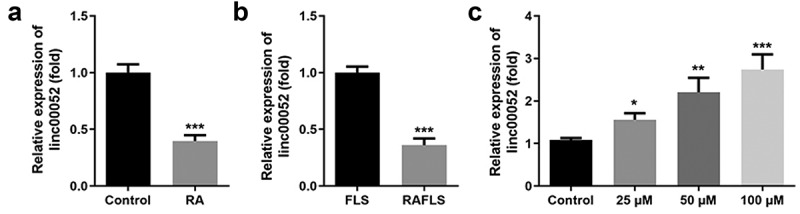
Expression of linc00052 is decreased in RA. (a) Relative expression levels of linc00052 are decreased in RA mice. (b) Expression levels of linc00052 are decreased in RAFLS cells. (c) Linc00052 levels were increased in RAFLS cells by curcumin treatment. *P < 0.05, **P < 0.01, ***P < 0.01 vs. Control. RAFLS, rheumatoid arthritis fibroblast-like synovial.

Linc00052 expression levels in RAFLS cells were determined following curcumin treatment. As shown in Figure 3(a,b), linc00052 expression levels were significantly decreased in the RA group in vivo and in RAFLS cells. Moreover, curcumin treatment increased linc00052 expression in a dose-dependent manner (Figure 3c).

### Silencing linc00052 reversed the effects of curcumin on the cellular functions of RAFLS cells

**Figure 4.**
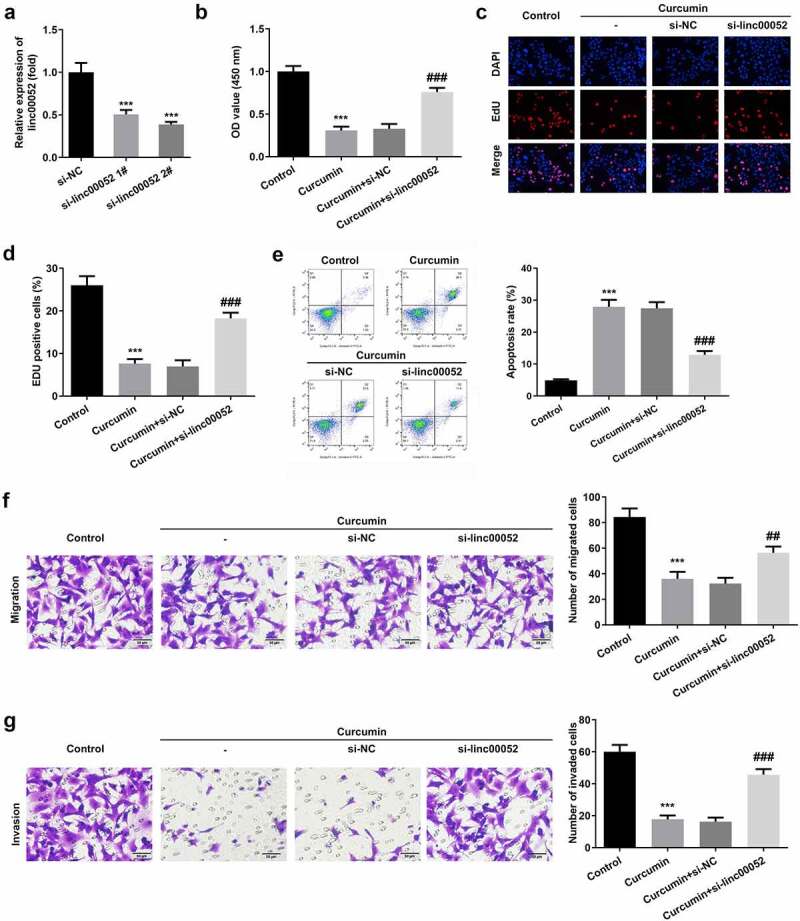
Knockdown of linc00052 reverses the effects of curcumin. (a) Transfection efficiencies of linc00052 in RAFLS cells. (b-d) Cell proliferation, (e) apoptosis, (f) migration and (g) invasion of RAFLS cells treated with curcumin and/or small interfering RNA of linc00052 (si-linc00052). ***P < 0.001 vs. Control. ##P < 0.01, ###P < 0.001 vs. Curcumin+si-NC.

Figure 4a shows the transcriptional efficiency of si-linc00052 (Figure 4a). Due to its high efficiency, si-linc00052 2# was used in subsequent experiments. As shown in Figure 4(b–d), linc00052 knockdown abrogated the effects of curcumin and promoted the proliferation of RAFLS cells. Linc00052 knockdown also induced the apoptosis of RAFLS cells (Figure 4e). Inhibition of migration and invasion of RAFLS cells induced by curcumin was antagonized by linc00052 knockdown (figure 4(f,g)).

### Linc00052 sponges microRNA (miR)-126-5p to regulate the mRNA expression levels of protein inhibitor of activated STAT 2 (PIAS2)

**Figure 5.**
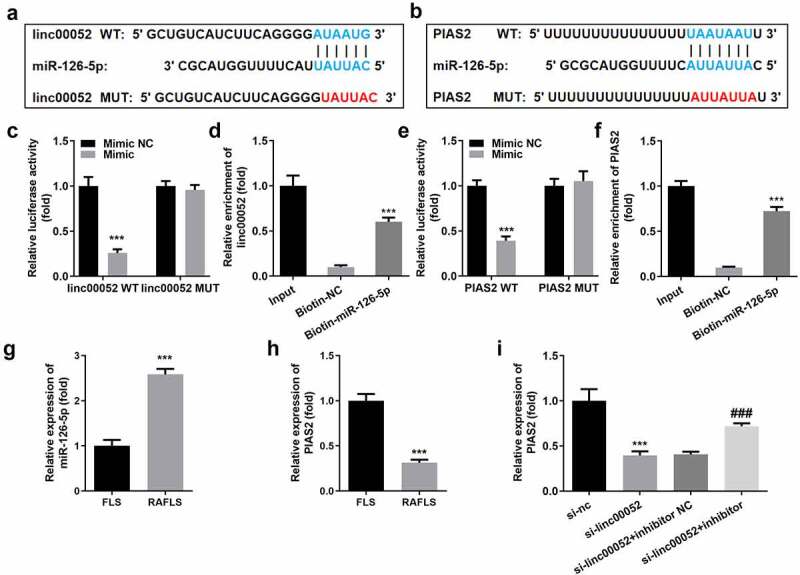
Linc00052 sponges microRNA (miR)-126-5p to regulate the protein inhibitor of activated signal transducer and activator of transcription 2 (STAT 2) (PIAS2). (a) Binding sites between linc00052 and miR-126-5p. (b) Binding sites between miR-126-5p and PIAS2. The interaction between linc00052 and miR-126-5p verified using luciferase (c) and RNA pull-down (d) assays. The interaction between miR-126-5p and PIAS2 verified using luciferase (e) and RNA pull-down (f) assays. (g) Expression levels of miR-126-5p in RAFLS cells. (h) Expression levels of PIAS2 in RAFLS cells. (i) Expression levels of PIAS2 in RAFLS cells after treatment with curcumin. ***P < 0.001 vs. Mimic NC, Biotin-NC, FLS, or si-NC; ^###^P < 0.001 vs. si-linc00052. CON, control; Cur, curcumin; RAFLS, rheumatoid arthritis fibroblast-like synovial.

LncRNAs function as competing endogenous RNAs (ceRNAs) to regulate gene expression by sponging miRNA(s). To further verify the underlying mechanisms, we explored the potential targets of linc00052. Figure 5(a,b) show that miR-126-5p binds to linc00052 and PIAS2. The interaction between miR-126-5p and linc00052/PIAS2 was confirmed using luciferase and RNA pull-down assays (Figure 5(c–f)). miR-126-5p was significantly overexpressed, while PIAS2 expression levels were downregulated in RAFLS cells (Figure 5(g,h)). Moreover, the decrease in PIAS2 expression levels induced by linc00052 knockdown was alleviated by the miR-126-5p inhibitor (Figure 5i).

### Overexpressed PIAS2 degrades the cellular functions of RAFLS cells

**Figure 6.**
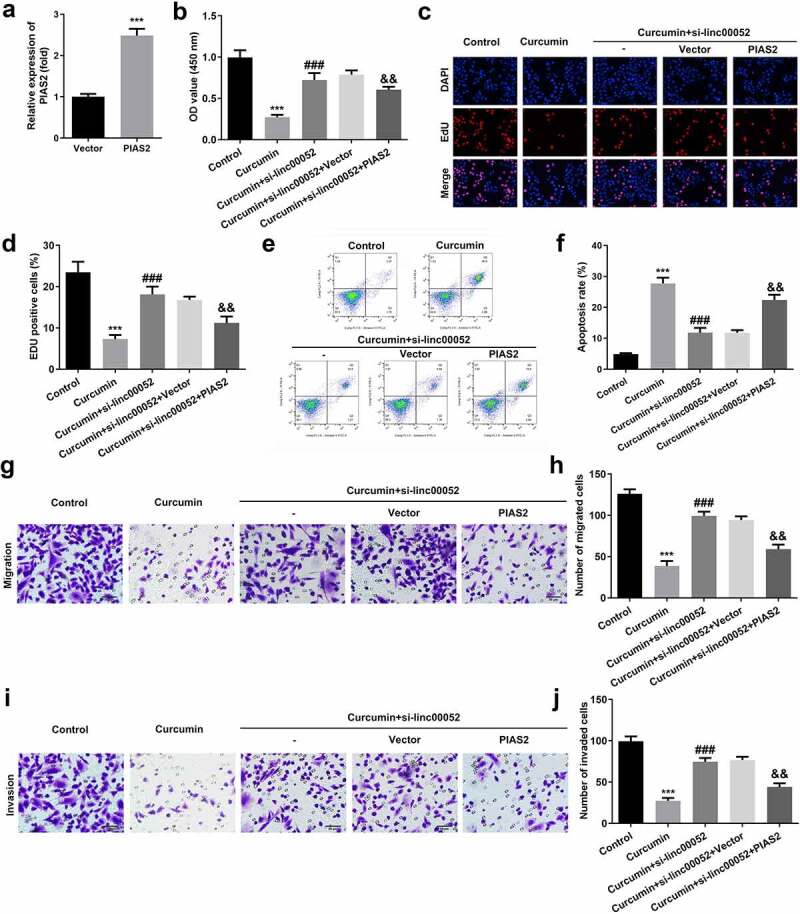
Knockdown of PIAS2 alleviates the effects of curcumin. (a) Transfection efficiency of PIAS2. (b-d) Cell proliferation, (e-f) apoptosis, (g-h) migration, and (i-j) invasion of RAFLS cells treated with curcumin and/or si-PIAS2. **P < 0.01 vs. CON or Vector; ^###^P < 0.001 vs. curcumin; ^&&^P < 0.01 vs. Curcumin + linc00052 + Vector. CON, control; Cur, curcumin; RAFLS, rheumatoid arthritis fibroblast-like synovial.

Figure 6a shows the transfection efficiencies of PIAS2 overexpression plasmids. Overexpression of PIAS2 markedly suppressed the cell viability and proliferation, while promoting the apoptosis of RAFLS cells (Figure 6(b–f)). Additionally, upregulation of PIAS2 expression remarkably inhibited the migration and invasion abilities of RAFLS cells (Figure 6(g–j)).

### Linc00052 knockdown induces the activation of Janus kinase 2 (JAK2)/signal transducer and activator of transcription 2 (STAT2) signaling pathway

**Figure 7.**
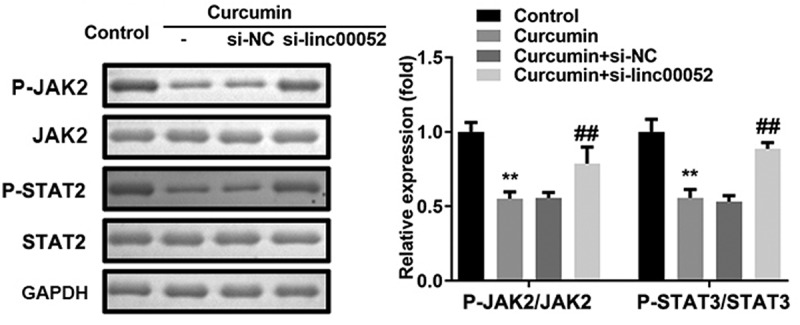
Linc00052 knockdown induces the activation of Janus kinase 2 (JAK2)/STAT2 signaling pathway. Protein expression of JAK2/STAT2 signaling. **P < 0.01 vs. Control; ^##^P < 0.01 vs. Curcumin + si-NC.

As shown in Figure 7, curcumin significantly decreased the protein expression levels of phosphorylated (p)-JAK2 and p-STAT3; however, linc00052 knockdown promoted the activation of the JAK2/STAT2 signaling pathway.

## Discussion

In this study, curcumin improved the structure of arthritis cells *in vivo* and induced the degradation of RAFLS cells. Additionally, curcumin suppressed RA development by regulating the linc00052/miR-126-5p/PIAS2 axis.

Curcumin is widely used in the treatment of many chronic diseases owing to its anti-inflammatory, antitumor, and antioxidant properties. A hydrophobic polyphenol derived from turmeric is a widely used cheap herb in China [25–27]. However, information regarding the use of curcumin for the treatment of RA is scarce. In the present study, we found that curcumin effectively inhibited RA both *in vitro* and *in vivo*. The pathogenesis of RA is complicated [2,28,29]. In RA, various inflammatory cells are activated that secrete pro-inflammatory cytokines, such as TNF-α and IL-1β, triggering a long-lasting inflammatory cascade [30] Additionally, overexpressed vascular growth factor and excessive proliferation of synovial fibroblasts lead to joint expansion and deformation of cells [31] Therefore, anti-inflammation and anti-proliferation are necessary for the treatment of RA. Sandeep et al. [32] found that curcumin induces the apoptosis and suppresses the proliferation and inflammation of psoriatic cells. Additionally, Li et al. [33] demonstrated that curcumin inhibits the proliferation and inflammatory response of angiotensin II-treated muscle cells. In this study, we revealed that curcumin improved RA development by promoting apoptosis and inhibiting the proliferation and inflammatory response of RAFLS cells. These results indicate that curcumin may be an effective drug for the treatment of RA.

LncRNAs are involved in the pathogenesis of RA [34]. Dysregulated lncRNAs in autoimmune and inflammatory diseases have limited contributions to the pathogenicity of RA. Linc00052 is involved in the regulation of cancer progression and inflammation [35]. However, the role of linc00052 in RA has not yet been reported. In our study, linc00052 expression levels were upregulated after curcumin treatment, whereas linc00052 knockdown reversed the effects of curcumin and exacerbated the development of RA. These results illustrate that linc00052 may be a potential target for the treatment of RA. LncRNAs function as ceRNAs to regulate gene expression by targeting miRNAs. For instance, lncRNA maternally expressed 3 exerts a protective role in RA by regulating the miR-141/serine-threonine kinase/mammalian target of rapamycin axis [32]. Long intergenic non-protein-coding RNA p53-induced transcript suppresses the proliferation and invasion of RAFLS cells by regulating the miR-155-5p/suppressor of cytokine signaling 1 axis [33]. In this study, linc00052 was found to function as a miR-126-5p sponge. miR-126 promotes the proliferation and inhibits the apoptosis of RAFLS cells. Moreover, miR-126-5p may be a biomarker for coronary atherosclerosis in patients with RA [34]. In the present study, miR-126-5p was found to be overexpressed in RA. Therefore, linc00052 suppresses RA development by regulating miR-126-5p.

PIAS, a suppressor of STAT signaling, participates in inflammatory responses, immune regulation, cell proliferation, and apoptosis. For instance, PIAS2 stabilizes SMAD4 to regulate transforming growth factor-β [35]. PIAS2 promotes papillomavirus replication by regulating papillomavirus helicase E1 protein [36]. Moreover, dysregulated PIAS2 expression induces the development of bone disorders [37–39]. In the present study, PIAS2 expression levels were found to be downregulated in mice with RA. Moreover, overexpression of PIAS2 induced the degradation of RAFLS cells and inactivated the JAK2/STAT2 signaling pathway. These findings suggest that the curcumin-induced upregulation of linc00052 expression suppresses the degradation and inflammatory response of RAFLS cells by regulating the miR-126-5p/PIAS2 axis.

## Conclusion

The results of this study revealed that curcumin suppressed the inflammatory response and synovial hyperplasia, while promoting the apoptosis of RAFLS cells via the linc00052/miR-126-5p/PIAS2 axis. These findings may aid in the development of a novel therapeutic strategy for the treatment of RA.

## Data Availability

The datasets used and analyzed during the current study are available from the corresponding author on reasonable request.
